# Pain Management Strategies before Pan-Retinal Photocoagulation for Diabetic Retinopathy: A Systematic Review

**DOI:** 10.1155/2024/6662736

**Published:** 2024-08-20

**Authors:** Mohammadkarim Johari, Mehdi Moallem, Abdulrahim Amini, Fatemeh Sanie-Jahromi

**Affiliations:** ^1^ Poostchi Ophthalmology Research Center Department of Ophthalmology School of Medicine Shiraz University of Medical Sciences, Shiraz, Iran; ^2^ Department of Ophthalmology School of Medicine Hormozgan University of Medical Sciences, Bandar Abbas, Iran

## Abstract

**Purpose:**

This systematic review aims to consolidate key findings regarding the efficacy of pain relief medications administered prior to pan-retinal photocoagulation (PRP) for diabetic retinopathy (DR).

**Methods:**

A comprehensive search of major databases from 1993 to 2023 was conducted. Clinical trials comparing pain relief drugs before PRP in patients diagnosed with DR requiring PRP treatment were eligible for inclusion. The assessment of pain scores involved the use of various scales, such as the visual analog scale (VAS), numerical rating scale (NRS), verbal rating scale (VRS), and other ordinal pain scales. In addition, laser parameters were taken into consideration during the analysis.

**Results:**

Twenty-two clinical trials from initial 150 studies were included in the review. Nine studies evaluated the pain relief effects of nonsteroidal anti-inflammatories NSAIDs (selective NSAID and nonselective NSAID), two studies compared the effects of opioids (conventional opioids and atypical opioids), and eleven studies investigated the effects of benzodiazepines, lidocaine, and other sedatives.

**Conclusion:**

This review synthesizes findings from multiple studies reporting pain as an adverse outcome of PRP in patients with advanced DR. Based on the evidence from reviewed clinical trials, the administration of lidocaine 2% via transconjunctival, retrobulbar, or peribulbar block along with specific NSAIDs, such as topical ketorolac administrated 24 hours before treatment or oral diclofenac potassium (50 mg) prior to PRP, demonstrated beneficial effects among patients with DR.

## 1. Introduction

Diabetic retinopathy (DR) is a well-known sight-threatening complication of diabetes mellitus (DM). Approximately 40% of the patients with diabetes over 40 years have DR to some degree [1]. Scatter, or pan-retinal photocoagulation (PRP) is the mainstay of treatment for diabetes-induced retinal sequela [2]. Generally, 1200–1500 laser spots (approximately 500 *μ*m in size) are applied to the retinal tissue. According to the Early Treatment Diabetic Retinopathy Study (ETDRS), in untreated patients with severe nonproliferative diabetic retinopathy (NPDR) or early proliferative diabetic retinopathy (PDR), the 5-year risk of severe vision loss is more than 50%. PRP is typically performed in an outpatient setting; the procedure is administered following pupillary dilation using mydriatic/cycloplegic medications and after a topical anesthetic is instilled. The laser energy is delivered via a biomicroscope or an indirect ophthalmoscope. The emerging laser beam is focused on the retina through a condensing lens, inducing a white retinal scar if applied effectively [3]. Despite emerging protocols and instruments in retinal laser treatment, the literature has reported that 64.1% of eyes receive laser treatment less than the recommended threshold, leaving a considerable portion of diabetic eyes undertreated [4]. This consequently increases the risk of visual loss. An important reason for this undertreatment is pain sensation during laser administration, preventing the continuity and efficacy required for successful management [5, 6]. In addition, pain may cause sudden inadvertent movement of the patient's eye during the procedure, increasing the risk of laser appliance-related complications [7]. So far, several analgesic strategies have been used to manage treatment better and relieve pain during PRP, with mixed results [8–11]. In the present study, we reviewed the clinical studies of palliative treatments during PRP in recent years and their clinical outcome. This article seeks to elucidate a more efficient pain management strategy in PRP treatment for DR.

## 2. Methods

**Figure 1:**
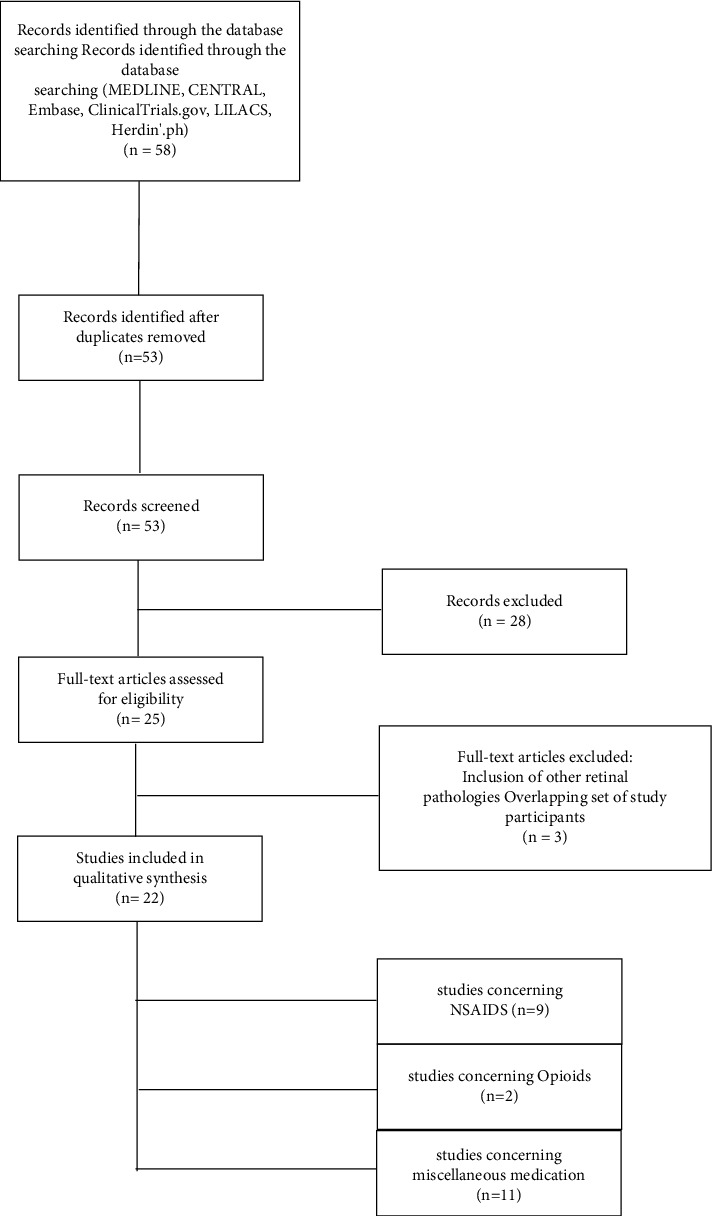
PRISMA flow diagram of included studies.

A systematic review was conducted to evaluate the efficacy of pain relief medications administered in conjunction with PRP in patients with PDR. To ensure accuracy and transparency in the records used for this study, the Preferred Reporting Items for Systematic Reviews and Meta-Analyses (PRISMA) guidelines were followed, as depicted in Figure 1.

### 2.1. Research Question and PICO Framework

The primary research question guiding this systematic review was “what is the effectiveness of pain relief medications administered before PRP in patients with DR?” This question was formulated using the Population, Intervention, Comparator, and Outcome (PICO) framework, which specifies the following elements:

 
*Population*: patients diagnosed with DR requiring PRP.
 
*Intervention*: administration of pain-relief medications before PRP.
 
*Comparator*: various pain-relief interventions, if available.
 
*Outcome*: assessment of pain scores using scales that include the visual analog scale (VAS), numerical rating scale (NRS), verbal rating scale (VRS), and other ordinal pain scales. Laser parameters (average laser power and the average number of laser spots) were also considered in the evaluation.

### 2.2. Inclusion Criteria

- Records that focused on the use of administration of pain relief medications before PRP.
- Studies on the diabetic patients who need PRP for treatment.
- Full-text papers; only randomized clinical trials (RCTs) and controlled clinical trials (CCTs).
- Journal articles published from earliest available time to 2023;
- No language limitation
- Primary sources with qualitative or quantitative research designs.

### 2.3. Exclusion Criteria

- Animal studies;
- Studies that focused on other retinal diseases or alternative laser therapies.

*Search Strategy and Reproducibility*: the search encompassed major databases, including Web of Science, PubMed, SCOPUS, MEDLINE, CENTRAL, EMBASE, and ClinicalTrials.gov. The following outlines the search criteria employed for pain relief medications before PRP. Inclusion criteria restricted articles to publications involving patients with PDR undergoing PRP. Exclusion criteria were articles focusing on other retinal diseases or alternative laser therapies unless separate data on PRP were provided.

Only RCTs and controlled clinical trials were considered in this study. Due to limited articles on this topic, no time limitation was applied, and the earliest year of publication for the articles was set to 1993, predating the writing of this paper in 2023. Only articles available as full texts were included.

### 2.4. Data Extraction

Two authors independently reviewed titles and abstracts of the search results. Data were extracted regarding the following keywords: “pain” AND “pan-retinal photocoagulation,” OR “retinal photocoagulation laser,” and “diabetic retinopathy” OR “proliferative diabetic retinopathy.” Alternate spellings, prefixes, and suffixes were also considered in the search strategy.

## 3. Result and Discussion

*Study Selection and Overview*. The total number of records identified through the database searches was 150, and the final number of articles used was 22, including both RCTs and CCTs (Figure 1). The studies were categorized based on the specific analgesic approach investigated.

*Categorization of Studies*. To enhance clarity and facilitate comparison, the refined data were further categorized based on the mechanism of the analgesic medicine investigated (Table 1).

Opioids (3.2.1);
(i) Conventional opioids
(ii) Atypical opioids
Nonopioids (3.2.2);
(i) Nonsteroidal anti-inflammatory drugs (NSAIDs)
  - Selective NSAID
  - Nonselective NSAID
(ii) Nonopioid miscellaneous analgesics
Benzodiazepines (3.2.3)
Amid local anesthetic (3.2.4)
Ca+ channel inhibitor (3.2.5)
Inhaled agent: entonox (3.2.6)

*Detailed Reporting of Medication Key Features and Clinical Outcomes*. In the subsequent sections, we present a detailed overview of each medication category, including key features and primary clinical outcomes. Refer to Table 1 for a comprehensive summary of these results.

*Discussion of Frequency and Comparative Analysis*. Throughout the results section, we aim to highlight the frequency of approaches observed in the different options studied, providing a detailed examination within each subcategory. The discussion following the presentation of individual medication categories will offer a comparative analysis, elucidating the place of each study within the broader context of analgesic strategies before PRP in patients with DR.

### 3.1. Pain and Pain Scale in PRP

Pain is a unique experience for an individual, perceived variably regarding the patient's age, sex, different cultures, experiences, and also the anxiety level. Hence, it would be challenging to compare the medication analgesic effects and pain perception across a group of patients [12].

Also, ocular pain elicited by argon laser photocoagulation is reported to be asymmetrically distributed in the retina, more intense in the upper temporal retina and periphery, notably in the 3 and 9 o'clock meridians of the retina that corresponds to the location of the long posterior ciliary nerves [13, 14].

Concerning a national survey held in the U.K., which included all units in the NHS ophthalmology medical training service, 88% of patients claimed that PRP could be painful. Such pain eventually led to distress and decreased patient compliance, reduced number of burns, and increased number of sessions in a significant fraction of treated patients. Interestingly, most centers involved did not have a pain control protocol reported, and oral analgesic use was reported in a minority of units [15].

Moreover, analgesia was used by the ophthalmologist in response to pain rather than preemptively [12]. It is noteworthy that it has been proposed that preemptive, compared to reactive analgesia, precludes central pain sensitization and local hyperalgesia, which in turn may increase the pain threshold during the procedure and reduce supplemental analgesic consumption [16, 17].

Pain described by patients included the following: sharp, piercing, intense, flashing, blinding, tiring, annoying, and pricking [18]. It is reported that adjusting laser device characteristics, such as reducing exposure time and increasing laser power, could cause less discomfort than the conventional single-spot method [6]. Furthermore, it is reported that patients undergoing secondary PRP claimed to perceive higher baseline pain than patients undergoing primary PRP [12, 19]. Although minimizing PRP-related pain may lead to a better administration of laser treatment, trials evaluating analgesia in treating PRP pain show variable results [20]. Such factors may account for mixed results and diversity observed in reported papers regarding PRP pain management. VAS (visual analog scale) is a commonly used scale in pain-related studies. It consists of a 10 cm scale, with numbers 0–10, arranged in deepening shades of gray, from light to dark. The patients are informed that 0 is no pain and 10 is the maximum pain they can perceive [21].

### 3.2. Analgesic Strategies

**Figure 2:**
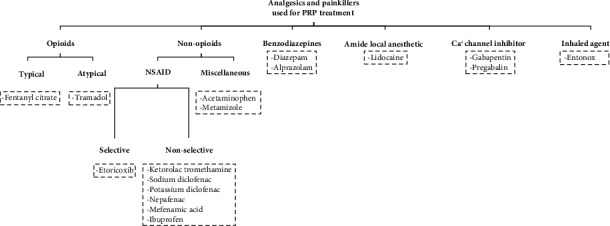
Categorization of the medications used for pain relief during PRP.

Various preemptive pain control agents with different mechanisms of action and administration methods have been studied to reduce PRP-related discomfort, including systemic use (oral or intramuscular injection), topical use, or retrobulbar/peribulbar injection. In addition to the reasons mentioned above, these methods would rather gain general acceptance or fall out of favor owing to different factors such as drug availability, ease of administration, systemic or local side effects, and even administration-to-effect interval [13]. Based on the reviewed studies, the drugs used for pain relief during PRP can be divided into 6 groups including opioids, nonopioids, benzodiazepines, amide local anesthetics, calcium channel blockers, and inhaled agents (Figure 2). Also, the most important trend has been toward the use of nonopioid (NSAIDs), which will be explained in detail in the following.

#### 3.2.1. Opioids

Opioid agents are potent and widely practiced in postop pain management in different fields. Opiate-related mechanism causes a reduction in neuronal excitability and inhibits the release of pain neurotransmitters. The molecular mechanism of action of opioid agonists is to cause hyperpolarization of neural cells after binding to their receptors. Opioid receptors are G protein-coupled receptors widely distributed in the brain and spinal cord, and to a lesser extent, in the peripheral nervous system and gastrointestinal system [22, 23]. Functionally, opioids can be divided into conventional (classic) and atypical opioids. The main difference between these two categories is that the effect of conventional opioids is based mainly on mu-opioid receptor agonism, whereas atypical opioids have multiple mechanisms of action and rely only partially on mu-opioid receptor agonism [24, 25]. The most common side effects of opiate use are nausea, vomiting, dizziness, constipation, psychological and physical dependence, and respiratory depression. However, their use has fallen out of favor in ophthalmology due to low safety profile and risk of abuse [26]. We extracted the studies reporting the use of conventional and atypical opioids for PRP pain relief.

*(1) Conventional Opioids.*

  Fentanyl Citrate
  Fentanyl citrate (FC) is a potent, short-acting conventional opioid analgesic. Rapid absorption and high tolerability make it one of the most utilized opioids. Hillier et al. evaluated oral transmucosal FC, an oral opioid agent in the form of a lozenge, as a one-dose premedication for PRP discomfort management. Systemic vital signs (pulse, blood pressure, respiratory rate, and pulse oximetry) were recorded on arrival, before lozenge consumption and after laser treatment completion at each visit. It was concluded that pre-emptive 200 *µ*g FC was effective as an analgesic for PRP pain management. In this study, patients receiving secondary PRP (1 week apart) tended to report higher baseline pain scores. However, patients undergoing secondary PRP also experienced the most significant reduction in the VAS pain score with fentanyl. Regarding vital signs, it was observed that this one-dose administration did not affect pulse, respiratory rate, or oxygen saturation to any statistically significant degree when compared with a placebo.
  Therefore, it was recommended as a relatively safe analgesic in an outpatient setting [19]. But there are drawbacks in this study as 9% of the patients experienced moderate to severe nausea and dizziness. Patients must remain supervised during and after treatment, which is inconvenient in an outpatient center. Also, considering the secondary physiological effects of FC, the process of the active intervention is likely to have been unintentionally unmasked to some patients, leading to some degrees of bias.

*(2) Atypical Opioids.*

  Tramadol
  Oral tramadol is an atypical opioid whose mechanism of action involves both opioid receptor activation and both serotonin and norepinephrine reuptake inhibition to achieve analgesia. Tramadol has advantages over other opioids, including a lower risk of respiratory depression or organ toxicity and lesser potential for abuse [27]. In a study, tramadol demonstrated no statistically significant superiority to the placebo. Nevertheless, in the tramadol group, fewer patients reported severe pain on the pain scale, and it was noted that tramadol effectively mitigated the rise in blood pressure during PRP sessions [28].

#### 3.2.2. Nonopioids

*(1) Nonsteroidal Anti-Inflammatory Drugs (NSAIDs)*. Among the most thoroughly studied analgesics in medical science are NSAIDs, which offer analgesic, anti-inflammatory, and antipyretic properties, and can be administered through systemic or local routes. They produce their clinical effects predominantly by inhibiting the cyclooxygenase (COX) enzyme, leading to a reduction in prostaglandin formation, a potent mediator of the pain pathway. The COX-1 subtype is expressed in many tissues, while the COX-2 subtype has limited inducible expression. Traditional NSAIDs inhibit both subtypes of COX-1 and 2, while certain products can exert more potent inhibition on either subtype, resulting in various efficacy and adverse reactions. The most common adverse reactions observed in systemic NSAID use are gastrointestinal toxicity, platelet dysfunction, altered renal function, and hepatic toxicity [29]. Ophthalmic topical NSAIDs application has shown good penetration and distribution throughout ocular tissue [30]. Although significant systemic absorption is demonstrated following topical ophthalmic administration, documented systemic side effects are rare. There are reports regarding exacerbation of bronchial asthma following topical NSAIDs use, which raised concerns about the safety of this medication [31–33]. It is assumed to be related to inhibiting the COX enzyme within the pulmonary system. Although there are no other reported clinical systemic reactions following topical NSAIDs application except for asthma exacerbation, the possibility of toxic systemic response should be kept in mind, particularly in prolonged use and application at extreme ages. Punctual occlusion intended to decrease systemic absorption and proper history taking and may alleviate this concern. Frequently observed local adverse events are hyperemia, foreign body sensation, contact dermatitis, local corneal anesthetic effect, and reports of corneal melting in prolonged use [34]. In addition, topical NSAIDs are used in the management of pain in the following conditions: corneal abrasions, inflamed pterygium, and pingueculitis. However, in posterior segment procedures, heterogeneous outcomes are encountered regarding the effects of NSAIDs [34–36]. It needs to be clearly established whether oral or topical NSAID products would result in higher concentrations at the level of the long posterior ciliary nerve in the suprachoroidal space, which is assumed to be the site of pain generation during PRP. It has been observed that in rabbit models, ketorolac, a widely used NSAID, can be found throughout the eye, including the choroid and retina after topical administration [37]. The topical form of indomethacin, another NSAID product, is shown to have higher aqueous levels than its oral form when used preemptively [38]. In the following, we reviewed the clinical reports on the use of selective and nonselective NSAIDs for the management of PRP pain.

Selective NSAID
    Etoricoxib
    Nascimento et al. evaluated analgesic effect of etoricoxib (Arcoxia®), a highly selective COX-2 inhibitor, during photocoagulation in patient with PDR and showed etoricoxib 120 mg reduces pain in the treatment group. The average oral bioavailability of etoricoxib is approximately 100%. Compared to nonselective COX inhibitors, it offers less gastrointestinal toxicity and is expected to have minimal effects on platelet function. Another advantage, mentioned in this study, is similar drug pharmacokinetics in the elderly compared to younger subjects and in patients with moderate to severe renal insufficiency compared to healthy subjects, making dose adjustments unnecessary [39, 40].
Nonselective NSAID
    Ketorolac Tromethamine
    Ketorolac, another commercially available NSAID product, is a nonselective COX inhibitor, reported as the most potent COX-1 inhibitor in the NSAID group. It is commercially available as a tromethamine salt that improves its water solubility and bioavailability. Systemic administration as an oral or intramuscular route has demonstrated 80–100% bioavailability, with an analgesic effect comparable to opioids [41]. Ketorolac tromethamine 0.5%, as a topical ophthalmic solution, has shown efficacy in reducing vitreous PGE2 levels in patients undergoing pars-plana vitrectomy [29]. Ketorolac tromethamine formulated at 0.4% was as effective as 0.5% solution for postcataract surgery inflammation control [42]. Topical ketorolac was assessed separately by Esgin and Samut [35] and Chewa Raja et al. [13] with different drop-to-treatment intervals. Esgin and Samut reported that topical ketorolac administrated 1 hour before treatment is no more effective than a placebo for pain relief during posterior segment laser procedures in PDR patients.
    On the other hand, Chewa Raja et al. concluded that this topical solution effectively alleviates pain in PRP treatment if applied 24 hours before PRP. It was postulated that although ketorolac tromethamine can achieve adequate maximal aqueous concentration within 1 hour, for an NSAID to reach maximal effectiveness, the existing endogenous supply of prostaglandins must be depleted, which cannot be reached within an hour predosing [37]. This was consistent with a previous study concluding that preoperative ketorolac tromethamine for 3 days or 1 day of predosing provided superior pharmacokinetic response compared to 1-hour pretreatment in cataract surgery [43]. This might explain why topical analgesics, particularly NSAIDs, show less than expected clinical efficacy despite proven in vitro effects although it cannot be overemphasized that such long waiting intervals may not be convenient in a daily outpatient practice. As an intramuscular injection (30 mg), systemic ketorolac was also studied by Wu et al. and showed no superiority to a placebo in PRP-related pain relief [11].
    Potassium/Sodium Diclofenac. Diclofenac is a potent NSAID used orally or topically, inhibiting COX cascade nonselectively. Apart from that, at higher doses, it also inhibits the lipo-oxygenase pathway, making it an excellent anti-inflammatory agent. Diclofenac topical solution contributes to more prominent and prolonged corneal anesthesia than other NSAIDs [44].
    In a trial conducted on 30 patients, two groups randomly received oral potassium diclofenac (50 mg) and a placebo sequentially, undergoing 2 PRP laser sessions in the same eye. The tablet was given 45 min before the PRP session since the peak plasma concentration of diclofenac is achieved between 20 and 60 minutes after oral administration. The pain was then assessed using a VAS chart. It was observed that the mean VAS pain score was lower in the diclofenac group [18]. This was consistent with a study by Zakrzewski et al. on 90 patients that analyzed the analgesic effect of oral diclofenac potassium (50 mg, 1 hour before PRP) and topical diclofenac sodium 0.1% compared to the control group. They demonstrated that oral diclofenac pretreatment in PDR patients provides statistically significant pain reduction compared to placebo during the PRP procedure. However, topical diclofenac instilled 1 hour before PRP showed no significant pain reduction [9]. Two nearly identical studies were published evaluating the efficacy of topical sodium diclofenac 0.1% for the management of PRP pain management in DR patients. Ramezani et al. [45] reported no statistically significant pain relief although the outcomes reported by Weinberger et al. [44] were statistically significant, particularly in female patients. It was proposed that this diversity in outcomes most probably resulted from different applied laser characteristics or drop-to-laser intervals; however, the mean laser setting and treatment interval were almost the same in both papers. It should be noted that even in the Weinberger et al.'s report, nine out of 45 perceived more significant pain using diclofenac than placebo and seven patients had similar pain levels using both treatments. Hence, these results may not be significantly different from the clinical point of view and must be interpreted cautiously.
    In another randomized prospective control study recently held by Aziz et al., topical sodium diclofenac 0.1% was administered to 90 patients 30–135 mins before PRP. Laser characteristics and drop-to-treatment interval were identical to the two previously mentioned studies. It was noted that pretreatment with topical sodium diclofenac 0.1% resulted in considerably lower pain levels than placebo, as reported by patients. This effect was more significant in the female group. However, 10 out of 47 patients still felt more significant pain while using diclofenac compared to the placebo and 8 had similar pain levels with both treatments [46].
    Nepafenac. Nepafenac 0.1% is an NSAID product in the form of ophthalmic suspension. It is a prodrug with significant corneal penetration, converted to the active metabolite, amfenac, a highly effective COX-2 inhibitor [47]. Nepafenac 0.1% was tested in PRP pain management and showed that it was effective for preemptive use in patients compared to the placebo, particularly in women. The results in men patients were not statistically significant [48].
    Mefenamic Acid. Oral mefenamic acid (500 mg), a widely used NSAID agent for pain management, was also studied by W-C Wu et al. and showed no superiority to the placebo in PRP-related pain relief [11].

*(2) Nonopioids Miscellaneous Analgesics.*

  Acetaminophen. Acetaminophen (APAP—also known as paracetamol in many countries) is a nonopioid analgesic and antipyretic agent used to treat pain and fever. Acetaminophen is a widely used analgesic with high availability and a well-established safety profile in therapeutic doses.
  However, Vaideanu et al., in their study found that pre‐emptive analgesia with paracetamol did not significantly reduce pain associated with PRP [20]. In another study by Johari et al., no evidence of the pain-relieving effect of combined 325 mg acetaminophen and 200 mg ibuprofen was found during PRP [49]. Wu et al. found that acetaminophen either alone or in combination with other analgesics (diazepam and mefenamic acid) was not effective in preventing PRP treatment-associated pain [9].
  Metamizole. Metamizole, also known as dipyrone, is an effective nonnarcotic analgesic and antipyretic agent which is assumed to inhibit prostaglandin synthesis. Despite concerns about reported hematologic adverse reactions, metamizole has yielded a well-documented safety profile in several studies [50]. Metamizole, ingested orally (1000 mg) 40 min before laser therapy, showed pain reduction in a randomized, double-masked clinical trial. Efficacy and availability in several countries make it an optimal pain reduction option. However, further studies are necessary to support this outcome since it was a small trial with a total of 21 patients [51]. In another study conducted in Brazil by Del Santo AM et al., oral metamizole (1000 mg) efficacy was compared to oral ibuprofen (600 mg), two commercially available NSAIDs. In this study, no statistical difference in the average pain level was reported between the 2 groups of patients. There was a tendency to feel severe pain with greater powers, but no significant difference existed [52].

#### 3.2.3. Benzodiazepines

Benzodiazepines are a class of psychoactive drugs that affect GABA-A receptor function by quickly diffusing through the blood-brain barrier and inducing central nervous system depression. Their rapid onset and immediate symptom relief make these compounds optimal for pain and discomfort reduction [53]. Oral diazepam (5 mg), oral diazepam with acetaminophen, and oral diazepam with mefenamic acid were evaluated by Wu W et al. regarding PRP pain reduction. Medications were ingested 2 hours before the PRP, and laser characteristics were similar statistically in each group. Treatment groups had no superiority in pain reduction over the placebo group [11]. This study is contrary to the results Muhammad Ali Haider et al. reported later, in which the combination of alprazolam (0.5 mg) and mefenamic acid (1000 mg) ingested 1 hour before the session was observed to be superior to no analgesia in PRP pain reduction.

However, one drawback of this study is that a placebo was not used in the nonmedication group [54].

#### 3.2.4. Amid Local Anesthetic

*Lidocaine*. Lidocaine, also known as lignocaine, is an antidysrhythmic and amide-based anesthetic utilized in a large variety of surgical procedures since 1948. It exerts anesthetic activity by blocking sodium channels of neurons in desired tissue, thereby inhibiting the propagation of neural stimulus. Injection, inhalation, or topical anesthetic application are different routes of lidocaine administration. However, an inadvertent intravascular injection in regional anesthesia may lead to severe cardiotoxicity, life-threatening arrhythmia, or CNS toxicity [55]. Different lidocaine compounds with various routes of administration are investigated for PRP analgesia. In a report, preemptive lidocaine 2% in the form of topical gel is reported to be effective in laser-related pain reduction. This is presumed to result from anterior segment pain fibers blockage either directly by contact with lidocaine gel or indirectly through the diffusion of the analgesic into the anterior chamber [56]. Furthermore, as previously stated in the abovementioned medication categories, Wu et al. performed a prospective, randomized study to determine the efficacy of various analgesic agents during PRP treatment [11]. A number of 220 patients were involved in this study. They were randomized into eight groups as follows: peribulbar lidocaine 2% injection (4 ml), oral diazepam (5 mg), oral acetaminophen (500 mg), oral mefenamic acid (500 mg), oral diazepam with acetaminophen, oral diazepam with mefenamic acid, ketorolac tromethamine (30 mg) intramuscular injection, and control group. Peribulbar anesthesia was administrated half an hour before treatment. Laser characteristics and spot number delivered were similar statistically in each group. First, this study demonstrated that PRP laser treatment is painful for most patients. Among the abovementioned drugs, only the peribulbar lidocaine 2% injection group had statistically significant lower pain scores. Also, it was noted that pain sensation could trigger a blood pressure rise. Such an increase in blood pressure is proposed to indicate hemodynamic responses to pain. Again, only peribulbar anesthesia was effective in mitigating such blood pressure rise.

Peribulbar injection in this study was performed through the lower and upper eyelids. Lower lid injection was immediately above the orbital rim at the junction of the medial two-thirds and lateral third, applied parallel to the orbital floor. The upper lid injection was immediately below the upper eyelid at the junction of the medial third and lateral two-thirds, applied parallel to the orbital roof. A 25 mm needle was used to perform the injection, introduced entirely through the skin. Peribulbar anesthesia was reported to have similar efficacy and a safer injection method than retrobulbar anesthesia, leading to fewer sight-threatening complications. Despite that, some severe ophthalmic and systemic complications have also been reported, such as hyphema, retrobulbar hemorrhage, globe perforation, expulsive hemorrhage, meningitis, and seizure [57]. Several options are available to reduce or prevent pain in patients who cannot effectively tolerate pain despite systemic or topical medication. Retrobulbar anesthesia is one of the most effective methods of analgesia. However, serious complications such as globe perforation, retrobulbar hemorrhage, traumatic optic neuropathy, central nervous system, and cardiorespiratory depression have all been described. Peribulbar and subtenon injections, on the other hand, carry more minor risks. In the 3 types of anesthesia injections mentioned above, retrobulbar injection is likely to cause deep orbital anesthetic spread, leading to varying degrees of akinesia. This is a disadvantage since ocular movement may enhance the view of the peripheral retina during laser application. These injection methods utilize complex techniques and have potential vision-threatening complications. Cuthbertson et al. reported that a 2.8 ml injection of lidocaine 2%, compared to a 5 ml injection, maintains kinesia while retaining good analgesia [58]. In a prospective randomized controlled trial on 65 patients, the analgesic effect of subconjunctival injection of lidocaine 2% was assessed. 0.5–1 ml of lidocaine 2% was injected with a 30-gauge needle 5 mm posterior to the limbus in the targeted quadrant of therapy. Patients who received anesthesia were 20 times more likely to be pain free than patients receiving placebo injections. No complications were described beyond subconjunctival hemorrhage, and this method is demonstrated to be safer than other injection methods [59].

Stevens et al. evaluated twelve patients in terms of anesthesia for PRP treatment using no-needle one-quadrant subtenon anesthesia. 1.5–2 ml of lidocaine 2% was delivered to the inferonasal quadrant in the subtenon space through an incision in conjunctiva made by scissors. Eleven out of the twelve patients had decreased pain scores. However, the delivery method versus placebo was subject to bias due to the placebo effect. It remains an invasive procedure, requiring an incision into the conjunctiva and tenon's fascia [60]. A mixture of bupivacaine 0.75%, lidocaine 2%, and hyaluronidase 3.75 IU/mL is also reported to alleviate pain in transconjunctival, retrobulbar, and peribulbar blocks [61]. However, patients included in this study received either argon laser PRP or krypton laser cyclophotocoagulation for glaucoma.

Also, a few patients were additionally administered intravenous fentanyl for anxiety control during the intervention, which was earlier mentioned to have analgesic effects in PRP patients independently.

#### 3.2.5. Ca^+^ Channel Inhibitor

*(1) Gabapentin/Pregabalin*. Gabapentin and pregabalin are structurally related compounds widely used in treating epilepsy and neuropathic pain. Gabapentin and pregabalin, already proven to be effective in alleviating early and late postoperative pain, are also evaluated in diabetic patients in terms of laser-associated pain reduction. In a previous study for management of PRP pain, two groups received gabapentin and pregabalin separately and were compared regarding the efficacy and adverse reactions. Pregabalin was noted to result in better rates of pain management. Pregabalin has a predictable and linear pharmacokinetics profile. Sedation, dizziness, nausea, and vomiting were adverse effects encountered in this study, with dizziness occurring in nearly 30% of the pregabalin group [62]. However, in another survey by Johari et al., pregabalin capsules (75 mg) did not reveal a significant difference in VAS levels and the patient's vital signs during PRP sessions [49].

#### 3.2.6. Inhaled Agent: Entonox

Another unique method of laser-related analgesia is inhaled agents. Entonox is a combination of 50% oxygen and 50% nitrous oxide. Inhalation of this mixture by the patient produces an analgesic effect without losing consciousness. Entonox has a good safety profile with few side effects. Cook et al. found that inhaled entonox is an effective analgesia method during PRP compared to a placebo, mainly when administered from the beginning of the laser session [12]. This study was the first systematic review that aimed to evaluate the effect of different pain relief medications in diabetic patients undergoing PRP due to DR. This review showed that patients who received lidocaine 2% injection (transconjunctival, retrobulbar, and peribulbar) had lower pain scores. Also, FC showed effective reduced pain during the PRP procedure. We faced mixed results regarding the effect of NSAIDs. Diclofenac, an NSAID used either orally or topically, is believed to provide significant pain reduction. However, topical use of diclofenac showed no significant pain reduction in several studies. Although, according to our review, a longer time interval between topical NSAIDs administration and the laser procedure might yield more potent effects in reducing pain during laser treatment, this review did not analyze the differences in the method of laser therapy in patients (conventional single-spot laser, conventional multi-spot laser, novel navigated laser), which seems to be effective in the patient pain sensation [6]. Also, the procedure's efficacy in managing advanced DR was not evaluated. It should be considered that the magnitudes of the pain sensation and scores should be interpreted cautiously, given that there were significant variations in the laser settings used.

Based on the evidence gathered in this systematic review, it is evident that effective pain management strategies for patients undergoing pan-retinal photocoagulation for diabetic retinopathy are crucial for enhancing patient compliance and comfort during the procedure. Our review highlights that the administration of lidocaine 2% via transconjunctival, retrobulbar, or peribulbar injection consistently results in significant pain reduction. In addition, topical NSAIDs, such as ketorolac, when administered 24 hours before the laser procedure, and oral NSAIDs, such as diclofenac potassium, taken one hour prior, also demonstrate beneficial analgesic effects. These findings suggest a strategic approach to pain management in PRP for DR; incorporating preemptive administration of NSAIDs within specific time frames and utilizing targeted local anesthetic injections. Implementing these protocols could potentially improve patient experience and adherence to PRP therapy, thereby enhancing the overall efficacy of DR management. Future research should aim to standardize laser settings and further investigate the differential impacts of various laser modalities on pain perception to refine these recommendations.

## Figures and Tables

**Table 1:** A summary of clinical trials applying analgesics for pain management during PRP.

Author/year	*n*	Fentanyl citrate	Tramadol	Etoricoxib	Ketorolac	Diclofenac sodium	Diclofenac potassium	Nepafenac	Mefenamic	Acetaminophen	Caffeine	Metamizole	Ibuprofen	Diazepam	Alprazolam	Lidocaine	Gabapentin	Pregabalin	Entonox	Route of administration	Laser (power (mW)/duration (s))	Effectiveness
Stevens et al., 1993	12															^*∗*^				StI	1000/0.15	Yes

Tong et al., 1997	65															^*∗*^				T		Yes

Weinberger et al., 2000	87					^*∗*^														T	500/0.1	Yes^1^

Cook et al., 2002	20			^*∗*^																Ih	—	Yes

Esgin et al., 2006	60				^*∗*^															T	600/0.13	No^2^

Wu et al., 2006	22													^*∗*^						O	450/0.2	No
	21									^*∗*^												
	21								^*∗*^													
	22									^*∗*^				^*∗*^								
	22								^*∗*^					^*∗*^								
	22				^*∗*^															II		
	23															^*∗*^				PI		Yes

Vaideanu et al., 2006	56									^*∗*^										O	—/0.1	No

Zakrzewski et al., 2009	90						^*∗*^													O	300/0.15	Yes
						^*∗*^														T		Yes

Hillier et al., 2009	35	^*∗*^																		O	—/0.05	Yes

Ko et al., 2009	28		^*∗*^																	O	—/0.15	No

Rodrigues et al., 2010	30						^*∗*^													O	500/0.15	Yes

Tesha et al., 2010	65															^*∗*^				ScI	500/0.15	Yes^3^

Nascimento et al., 2012	44			^*∗*^																O		Yes

Castro et al., 2014	30							^*∗*^												T	—/0.1	Yes^4^

Hazem et al., 2014	60																^*∗*^	^*∗*^		O	600/0.1	Yes^5^

de Araújo et al., 2015	21											^*∗*^								O	350/0.2	Yes

Santo et al., 2016	34											^*∗*^								O		No
													^*∗*^									

Ramezani et al., 2017	100					^*∗*^														T	900/0.2	No

Chewa et al., 2021	33				^*∗*^															T	200/0.1	Yes

Haider et al., 2021	560									^*∗*^					^*∗*^					O	—/0.1	Yes

Aziz et al., 2022	90					^*∗*^														T	100–500/0.1	Yes^6^

Johari et al., 2023	60									^*∗*^	^*∗*^		^*∗*^							O	∼400/0.15	No
																		^*∗*^				
										^*∗*^	^*∗*^		^*∗*^					^*∗*^				

Ih: inhalation, II: intramuscular injection, n: number of patients, O: oral, PI: peribulbar injection, ScI: subconjunctival injection, StI: subtenon injection, T: topical. ^1^Nine out of 45 patients had more pain with sodium diclofenac. ^2^More pain in females. ^3^Less anatomic complications than subtenon/retrobulbar injections. ^4^Effective in women. ^5^Effective in the pregabalin-treated group, the pregabalin-treated-group experienced more sedation and dizziness. ^6^More significant in females, 10 of 47 patients had more pain with diclofenac.

## Data Availability

The data used to support the findings of this study are available from the corresponding author upon request.
